# Providing Outstanding Undergraduate Research Experiences and Sustainable Faculty Development in Load

**DOI:** 10.3389/fpsyg.2019.00196

**Published:** 2019-02-11

**Authors:** Katherine R. Mickley Steinmetz, Alliston K. Reid

**Affiliations:** Department of Psychology, Wofford College, Spartanburg, SC, United States

**Keywords:** research in teaching load, sustainable research, publishing with undergraduates, faculty professional development, research opportunities

When budgets are limited and teaching loads are high, colleges and universities often face challenges to provide opportunities for faculty development and superior undergraduate research experiences. However, conducting research in one's field and allowing undergraduates to engage in this research can deeply enrich the experience of both professors and students (Kuh et al., 2007; Kuh, 2008). Therefore, the psychology department at Wofford College solved these problems by incorporating research into most psychology courses (especially lab courses) and by designing a laboratory experience which includes original team-based research designed for publication, all within the normal faculty teaching load. This fits with our departmental learning goals, stressing the scientific method and reliance on empirical research in the development and testing of psychological theories. This unique structure of our department, including research in load, gives a double benefit: (1) enhancing the ability for professors to continue research in their area, and (2) allowing students to engage in publishable research. We describe how we implemented these opportunities, hoping that some readers might adopt features into their programs.

## Research Opportunities

Wofford College is a 4-year, residential, undergraduate liberal arts college. The psychology program graduates 25–44 majors each year (*M* = 33) with seven full-time professors. The psychology department prepares majors to produce publication-quality research by providing three types of research opportunities: (a) The apprenticeship-based Senior Thesis required of all majors; (b) The research-team core course (RTCC) in which classmates work as a research team during the laboratory component to carry out a single large-scale experiment designed for publication; and (c) Independent studies which provide additional opportunities for publication-quality research in various labs throughout the academic year. Because these independent studies are commonly provided by many schools, this article focuses on the novel characteristics of the first two categories: the senior thesis and the RTCC, with their strong records of undergraduate co-authorship with professors.

## The Research Team Core Course (RTCC)

Wofford's psychology department prepares its majors to produce peer-reviewed research by incorporating and progressively building upon research methods in most courses offered in the major. Empirical research, data analysis, critical thinking, and writing begin in their first introductory lab course and advance continually through their senior year (National Research Council, 2000; Kuh, 2008). Nevertheless, the Research Team Core Course (RTCC) is unique.

While the classroom provides the core course material commonly expected in most psychology programs (such as Learning, Sensation and Perception, or Cognitive Psychology), its laboratory component organizes upper-level students to work together on a single novel experiment designed for publication.

Learning and Adaptive Behavior is a required semester-long lab course offered once or twice each year by one of the authors [AKR] with around 24 students. We realized that our students would benefit more from the active, hands-on critical-thinking approach provided by original, verifiable, scientific research as a research team than they would by exposure to demonstration labs that are widely provided as research experiences (National Research Council, 2000; Kuh et al., 2007; Kuh, 2008; Brown et al., 2014). In creating this RTCC, we replaced all demonstration labs with one *original* semester-long experiment with rats, without altering program requirements for the major. We incorporated original publication-quality research as an active critical-thinking approach, so experiments are new every semester—not even the professor knows what the results will be [(Boyer, 2004); *Scholarship of Discovery*]. To prepare for the course, the professor must design a unique publication-quality experiment that can be completed in the time available (before Thanksgiving or Spring Break). Because this RTCC relies on computers and rats, the professor must obtain IACUC approval and the rats, and write the new computer programs for all training and experimental conditions before the course begins. The background reading for each experiment changes each semester, but by focusing on a consistent research topic across years, students begin by reading the relevant articles produced by previous students. These articles demonstrate the progression of scientific knowledge produced by students previously in this course, and students then integrate this research with other published articles on the same general topic (*Scholarship by Integration*). We identify several of these articles in the reference list below.

We ask all RTCC students to work in two-person teams as the class carries out the major research project. Each team splits the daily responsibilities for running their two rats, yielding 3–4 h per week per student. Each team writes a major research report in APA style in which they analyze and describe data from all subjects in the experiment. They also describe the data from their two rats to evaluate consistency across the different teams. By writing their graded research reports, students learn the role of statistical inference while working with data that they collected, and how to work both as a two-person team and as part of a larger research team. Writing their research report helps them learn how their research may create new knowledge in the field, and structured feedback strongly emphasizes appropriate ways of explaining their scientific research to other scholars (*Scholarship of Teaching: Public Dissemination*).

A self-selected group of 3–5 students from the course represents the class as they present this research in a formal oral presentation at the Wofford College Science Research Symposium held at the end of each semester. We normally invite these students to become coauthors of the manuscript that the professor and students prepare together and submit after the course is over. Of course, not every experiment results in publication, but many do. To achieve publication, the professor may need to dedicate part of the following summer to writing and revising the manuscript with students (those willing to do the extra work of earning co-authorship after the course is over). This design has been highly successful and exposes these students to the rigors of the professional peer-review process. The course requires hard work, but students proudly speak about this experience for years. While the professor must put in substantial effort before and after the semester, this effort is usually not an increase in the steps needed for the professor to do this research as normal professional development outside of the course context. Professors could also involve students in the preparation of the RTCC experiment by offering elective course credit in experimental design.

Developing these RTCC courses is one way for the teacher/scholar to produce publication-quality research with student co-authors within the professor's normal teaching load and have the research funded by the teaching budget (“teaching through research mentoring”). Various designs of this RTCC can be implemented in any core laboratory course in the major, and the implementation details would vary across topics. We have offered this single RTCC as a program requirement for about 10 years, continually making improvements and working out the kinks. Now that we know that this course is highly effective, other Wofford psychology professors teaching core lab courses have the option and flexibility of including similar team-based research into their labs, creating their own RTCC. Naturally, this option is also available to readers of this paper and can be modified for different institutions, such as students becoming participants during class time, when class sizes are large or at institutions with many commuter students.

## The Apprenticeship-Based Senior Research Thesis

The senior thesis often represents the capstone of a student's education in psychology. These sorts of culminating experiences have been shown to be a fruitful learning tool which also makes students attractive to future employers (Kinzie, 2013; Budwig and Jessen-Marshall, 2018). Liberal-arts colleges may offer a thesis option in many forms: (a) It may be available only for “honor” students in the department, offered to any interested student, or required for every major. (b) Students may work as a team, or students may have their own individual thesis project. (c) The thesis may last one semester or longer. (d) The thesis may require empirical research, data analysis, and a complete research report; or it may be limited to a review of the research literature and the design of an interesting experiment (without carrying out that experiment). These options have important implications on faculty load, the quality of the thesis project, and subsequent prospects for publication.

Given these considerations, the psychology department at Wofford College requires a one-semester apprenticeship-based senior thesis to be completed by every psychology major. The RTCC and other laboratory experiences provide an excellent background to carry out and write a publication-quality senior thesis. Each of our seven full-time psychology professors mentors a small research team every academic year. This senior thesis is considered a laboratory course as part of the faculty credit load. By working in teams of 3–6 students, the daily responsibilities for carrying out the research project (experiment, data analysis, and research report) can be shared, which allows larger, higher-quality projects designed for publication to be completed within the timeline. Each professor designs the thesis project in his or her field of study and prepares for the research to start as the semester begins. The research team collaborates on all aspects of the research report and presents their research in a formal, college-wide oral presentation at the Science Symposium held at the end of each semester.

A second component of the senior thesis generally occurs in the first half of the semester while students are running the experiment(s)—the integrated review of the research literature written individually by each student (*Scholarship by Integration*). Under the professor's guidance, each student selects a topic for this comprehensive literature review. Depending upon the professor, the topic may be directly related to the research project or more related to the student's career goals—but the review must integrate scientific research published in journals. Each student submits the literature review for feedback as a graded APA-style paper (averaging 30–40 pages) in the format of *Psychological Bulletin*. All literature reviews and thesis reports are maintained indefinitely in the department archives. Students often include copies of both papers when they apply to graduate school. The combination of the group research project and the individually written literature review allows students to experience the two unique learning opportunities, completing a project that is entirely independently conceived (the literature review) along with one that is conducted as part of a team (the research project).

After the semester is over and students have presented their research at the symposium, the professor decides whether the research is appropriate for publication or whether more research is needed next year. Professors often encourage these students to become coauthors of the manuscript, conference presentation, or poster that the professor and student co-authors prepare and submit after the semester is over. Some graduating students opt out. Though this differs from many traditional senior thesis programs where each student conceives of his or her own project, this design allows *every* student to engage in high-quality research and to receive an outstanding educational experience. Every psychology professor shares the same responsibilities and opportunities for faculty development, no matter what their field of study.

## Conclusion

The beauty of the RTCC and the Apprenticeship-Based Senior Thesis is that both are considered laboratory courses. Thus, funds that are set aside for the annual teaching budget can be used to conduct research within the faculty member's research area as part of the laboratory course. As a result, financial resources for all lab courses have been available reliably every semester. Much of this research is submitted for publication with student coauthors in peer-reviewed journals under the professors' guidance and contributes to the professor's professional development. Including the RTCC and Senior Thesis as part of the curriculum allows a unique opportunity to give undergraduates outstanding research experiences, while allowing professors to thrive within their own area of study without increasing their teaching load. This model could be adapted to different types of institutions as the data collection takes place in the context of a course. We have found since implementing this curriculum that while teaching loads did not increase, *every* psychology professor has continual faculty development opportunities, and *every* psychology major is involved in empirical research with opportunities to coauthor research published in high-quality peer-reviewed journals.

## Author Contributions

All authors listed have made a substantial, direct and intellectual contribution to the work, and approved it for publication.

### Conflict of Interest Statement

The authors declare that the research was conducted in the absence of any commercial or financial relationships that could be construed as a potential conflict of interest.

## Notes

Here are some recent articles with student coauthors from my RTCC on Learning and Adaptive Behavior. Each article contains one or more of our experiments related to skill learning—the central theme of our research. RTCC students read these articles in chronological order to understand the research, the evolving research questions, and how they (too) can become coauthors.—AKR.

Reid, A. K., DeMarco, G., Smith, K., Fort, T., and Cousins, E. (2013a). The influences of guiding cues on motor skill autonomy in rats. *Learn. Behav*. 41, 455–463. doi: 10.3758/s13420-013-0121-y

Reid, A. K., Folks, N., and Hardy, J. (2014). On the dynamics of stimulus control during guided skill learning in nonhumans. *Behav. Process*. 104, 72–83. doi: 10.1016/j.beproc.2014.01.017

Reid, A. K., Futch, S. E., Ball, K. M., Knight, A. G., and Tucker, M. (2017). Assessment of progressively delayed prompts on guided skill learning in rats. *Learn. Behav*. 45, 62–75. doi: 10.3758/s13420-016-0241-2

Reid, A. K., Nill, C. A., and Getz, B. R. (2010). Changes in stimulus control during guided skill learning in rats. *Behav. Process*. 84, 511–515. doi: 10.1016/j.beproc.2010.01.001

Reid, A. K., Rapport, H. F., and Le, T.-A. (2013b). Why don't guiding cues always guide in behavior chains? *Learn. Behav*. 41, 402–413. doi: 10.3758/s13420-013-0115-9

Here are some articles that have come out of the apprenticeship-based senior thesis from my lab, all examining the effects of stress and emotion on memory.—KRMS

Mickley Steinmetz, K. R., Anderson, A., Brasher, K., and Brehmer, T. (2017a). Cortisol and stimulus-induced arousal level differentially impact memory for items and backgrounds. *Cogn. Emot*. 31, 325–338. doi: 10.1080/02699931.2015.1111197

Mickley Steinmetz, K. R., Knight, A. G., and Kensinger, E. A. (2016). Neutral details associated with emotional events are encoded: evidence from a cued recall paradigm. *Cogn. Emot*. 30, 1352–1360. doi: 10.1080/02699931.2015.1059317

Mickley Steinmetz, K. R., Sturkie, C. M., Rochester, N. M., Liu, X., and Gutchess, A. H. (2017b). Cross-cultural differences in item and background memory: examining the influence of emotional intensity and scene congruency. *Memory* 26, 751–758. doi: 10.1080/09658211.2017.1406119
